# Supplementary material for: Gastric Cancer Extracellular Vesicles Tune the Migration and Invasion of Epithelial and Mesenchymal Cells in a Histotype-Dependent Manner
Source: Int J Mol Sci. 2019 May 28;20(11):2608. doi: 10.3390/ijms20112608 (PMC6600627; doi:10.3390/ijms20112608)
Supplement: Supplementary file 1 [file ijms-20-02608-s001.pdf]

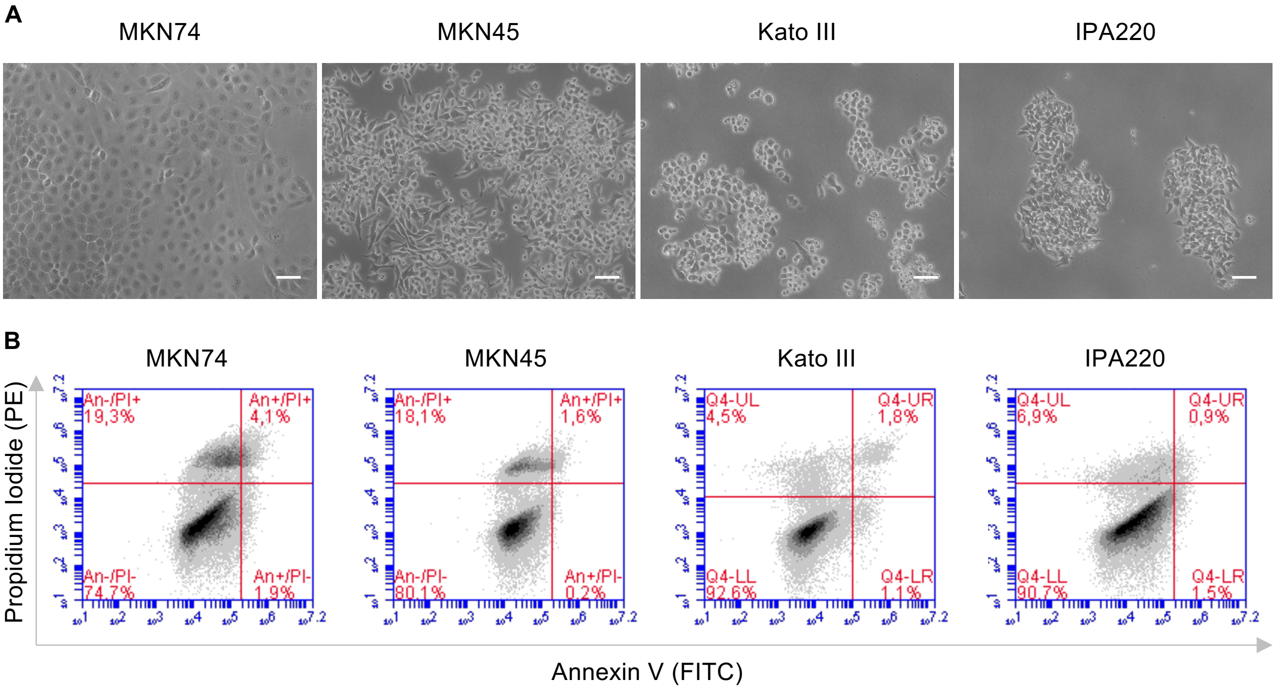

Figure S1. Characterization of GC cells before EVs isolation. (**A**) Representative bright-field microscopic images of MKN74, MKN45, Kato III and IPA220 GC cell lines. MKN74 and MKN45 displayed a more differentiated morphology, resembling the intestinal-type GC. Kato III and IPA220 presented a poorly differentiated phenotype, representative of diffuse-type GC. (**B**) Viability of GC cells tested by flow cytometry of annexin V (FITC) and propidium iodide (PE).

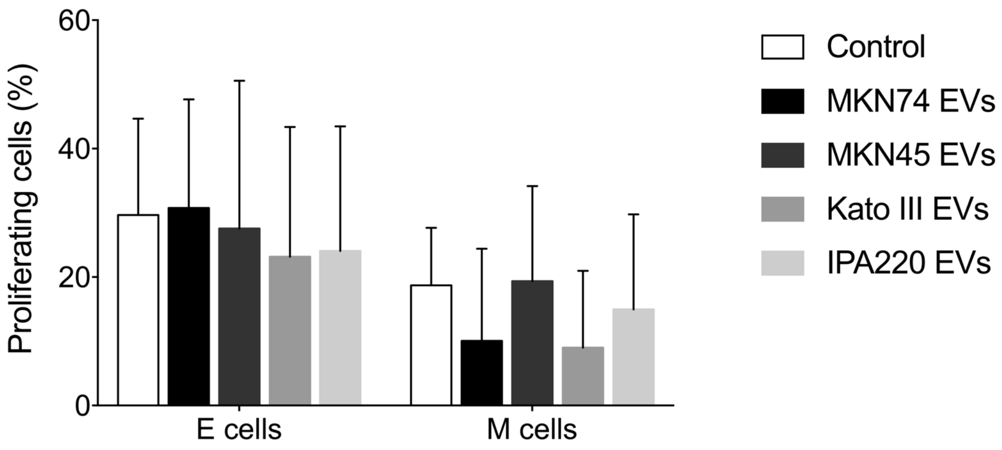

Figure S2. GC-EVs do not affect proliferation of epithelial and mesenchymal recipient cells. Graphs represents the mean percentage of proliferating cells ± standard deviation of three independent experiments.
